# Preclinical Imaging for the Study of Mouse Models of Thyroid Cancer

**DOI:** 10.3390/ijms18122731

**Published:** 2017-12-16

**Authors:** Adelaide Greco, Luigi Auletta, Francesca Maria Orlandella, Paola Lucia Chiara Iervolino, Michele Klain, Giuliana Salvatore, Marcello Mancini

**Affiliations:** 1 Dipartimento di Scienze Biomediche Avanzate, Università degli Studi di Napoli Federico II, 80131 Napoli, Italy; adegreco@unina.it (A.G.); micheleklain@libero.it (M.K.); 2 Istituto di Biostrutture e Bioimmagini, Consiglio Nazionale delle Ricerche—IBB, CNR, 80145 Napoli, Italy; direttore@ibb.cnr.it (M.M.); 3 CEINGE Biotecnologie Avanzate s.c.ar.l., 80131 Napoli, Italy; 4 IRCCS S.D.N., 80134 Napoli, Italy; francescaorlandella@libero.it (F.M.O.); paolalc89@libero.it (P.L.C.I.); giuliana.salvatore@uniparthenope.it (G.S.); 5 Dipartimento di Scienze Motorie e del Benessere, Università di Napoli Parthenope, 80133 Napoli, Italy

**Keywords:** thyroid cancer, preclinical imaging, ultrasound, nuclear medicine, theranostic, mouse models

## Abstract

Thyroid cancer, which represents the most common tumors among endocrine malignancies, comprises a wide range of neoplasms with different clinical aggressiveness. One of the most important challenges in research is to identify mouse models that most closely resemble human pathology; other goals include finding a way to detect markers of disease that common to humans and mice and to identify the most appropriate and least invasive therapeutic strategies for specific tumor types. Preclinical thyroid imaging includes a wide range of techniques that allow for morphological and functional characterization of thyroid disease as well as targeting and in most cases, this imaging allows quantitative analysis of the molecular pattern of the thyroid cancer. The aim of this review paper is to provide an overview of all of the imaging techniques used to date both for diagnosis and theranostic purposes in mouse models of thyroid cancer.

## 1. Introduction

Thyroid cancer is a common endocrine malignancy with increasing incidence worldwide, especially in women [1,2]. Carcinoma derived from thyroid follicular cells includes well-differentiated papillary (PTC) and follicular (FTC) thyroid carcinoma, poorly differentiated (PDC) thyroid carcinoma and undifferentiated or anaplastic thyroid carcinoma (ATC). PTC, which represents the most common cancer of the thyroid gland, generally has a good prognosis, although some patients develop metastases and are refractory to therapy [3,4,5]. FTC is the second most common thyroid malignancy and presents a 5-year survival rate of approximately 90%, although in some cases, the tumor can metastasize to the bones and lungs [3,4,5]. ATC is an inoperable tumor that is refractory to conventional therapies with a mean survival time from diagnosis of approximately 6 months [3,4,5,6]. PDC includes a heterogeneous group with an intermediate clinical behavior between well-differentiated carcinoma and ATC [4,7]. Medullary thyroid carcinoma (MTC) originates from neural-crest derived thyroid C cells; it is a rare and aggressive tumor that can present either sporadically or in a hereditary form [8].

Recently, through a whole genome sequencing approach, the Cancer Genome Atlas elucidated the genetic of PTC [9]. Different driver mutations encompassing genes that signal through the mitogen-activated protein kinase (MAPK) are associated with different histologic variants of PTC and the mutations are mutually exclusive [4,5,9]. The BRAF V600E mutation accounts for the vast majority of PTC cases; *RAS* mutations and gene rearrangements of the tyrosine kinase receptor *RET* are also genetic alterations frequently found in PTC [9]. In addition, rearrangements of *ALK*, *NTRK*, *FGFR2*, *MET*, *LTK* and *THADA* and mutations of *EIF1AX*, *PPM1D*, *CHEK2* genes have been reported with lower frequency in PTC [9]. FTCs instead are mainly characterized by *RAS* mutations and *PAX8/PPARγ* rearrangements [10,11,12]. Mutations that activate both MAPK and phosphoinositide 3-kinase (PI3K) signaling pathways are found in PDC and ATC [7]. Furthermore, ATC harbors additional mutational events in the tumor suppressor *p53*, in the *TERT* promoter and in the *CTNNB1* gene [6]. MTC instead is caused mainly by *RET* point mutations, whereas only a small proportion of cases are caused by *RAS* mutations [13].

Given the significant recent advances in the understanding of thyroid cancer biology, it is fundamental to exploit imaging techniques in thyroid cancer mouse models that can evaluate novel therapeutic strategies.

Several mouse models of thyroid cancers have been developed in recent years to understand the molecular mechanisms involved in thyroid tumorigenesis and to discover possible novel target therapies. To date, there are many different approaches available for imaging thyroid cancer in mouse models. This review is a comprehensive summary of all imaging techniques used for the molecular characterization of thyroid carcinoma in mice models.

## 2. Mouse Models of Thyroid Cancers

Following is a brief description of the principal mouse models of thyroid cancer including transgenic, xenografts, orthotopic and metastatic mouse models; the models used in the preclinical imaging studies are a focus of this review.

### 2.1. Transgenic Mouse Models

Transgenic mice are genetically engineered to introduce specific cancer-associated mutations into their genome, including activated oncogenes or loss of tumor suppressors (“knockout” strategies). The aberration is under the control of a specific promoter in the cells of a particular tissue, making the mouse prone to developing cancer [14]. The transgenic mouse model presents the advantage of faithfully recapitulating human disease. Another major advantage of this technique is represented by the possibility to use the model constitutively or conditionally. Since some cancer-altered genes are incompatible with life in mice, in the conditionally genetically engineered mouse, the expression of the gene is closely controlled both spatially and temporally [15]. Nevertheless, the transgenic mouse model has the disadvantages of a high cost of establishment and maintenance and the length of time needed to develop the tumors [14]. Given the main genetic lesions identified in thyroid carcinoma, several transgenic mouse models of thyroid cancer have been developed [16,17,18]. Following is a brief description of the transgenic mouse models of thyroid cancers used in the preclinical imaging studies reviewed in this article.

#### 2.1.1. BRAF V600E Transgenic Mouse Model

Given the prevalence and the important prognostic role of the BRAF V600E mutation [19], several mouse models expressing this transgene in the thyroid were developed. Initially, Knauf and colleagues generated a mouse model of PTC with thyroid-specific expression of BRAF V600E through the bovine *thyroglobulin (TG*) promoter [20]. These mice present with goiter and invasive PTC, reflecting the human PTC phenotype, although distant metastases were absent. In particular, the thyroid glands of the *TG-BRAF* mice were enlarged by 5 weeks of age, while PTCs were present at 12 and 22 weeks [20]. Since the expression of the *BRAF* oncogene tends to induce dedifferentiation and thus loss of the transgene expression, a doxycycline-inducible BRAF V600E mouse model (*Tg-rtTA/Tet/O* BRAF V600E) was generated. The inducible expression of oncogenic BRAF V600E in thyroid cells activates MAPK signaling and induces an invasive PTC. On the other hand, PTC regressed upon doxycycline withdrawal and the normal thyroid follicular architecture was re-established [21]. Thereafter, several other BRAF V600E transgenic mouse models were developed and all the different models generated showed a similar phenotype [16,17].

#### 2.1.2. *TRK-T1* Transgenic Mouse Model

*TRK1-T1* is an oncogene formed by the fusion of the *TPR* and *TRK* genes that are frequently found to be activated in PTC [9]. Russell et al. generated a transgenic mouse model in which *TRK-T1* is targeted in the thyroid through the bovine *TG* promoter. These mice developed hyperplasia by 7 months of age and/or a carcinoma without metastasis [22]. The tumors were not associated with inflammation, which is characteristic of human PTC [23].

#### 2.1.3. *TRβ-PV* Transgenic Mouse Model

Thyroid hormone receptor β (THRB) is a nuclear protein that regulates cell proliferation, differentiation, apoptosis and the release of thyroid hormones [24]. A mouse model of FTC was induced by the introduction of a dominant negative mutation in the *THRB* gene (*TRβ^PV/PV^*). The mutation caused a loss of transcriptional activity of T3 hormone due to a loss of THRB binding. Because of the lack of negative feedback by T3, TSH was upregulated and follicular cell hyperplasia and FTC were observed in the mouse thyroids [25]. The mice also showed lung and heart metastases by the age of 5 months [26,27].

#### 2.1.4. *Rb*^+/−^ Transgenic Mouse Model

The retinoblastoma gene (*Rb*) is a tumor suppressor and master regulator of the cell cycle. Inactivating mutations of *Rb* have been found in many human cancers [28]. To understand the role of *Rb* in the onset of tumors, several mouse models were generated. In mice heterozygous for *Rb* (*Rb*^+/−^), the onset of thyroid C cell hyperplasia that evolved to MTC was observed and the tumors were small and non-invasive [18,29,30,31].

### 2.2. Xenograft and Orthotopic Mouse Models of Thyroid Cancer

Apart from genetically engineered mice, the administration of carcinogenic substances and the direct implantation of patient derived cells are two methods to obtain in vivo growth of thyroid carcinoma in animal models [32]. Whereas the former system relies on the goitrogenic effect of various chemicals and leads to inconsistent and infrequent development of thyroid neoplasms, the latter has been successfully used in recent decades to study various aspects of thyroid tumorigenesis and response to therapies [32,33]. In particular, both the subcutaneous, i.e., xenograft and the intrathyroidal, i.e., orthotopic, injection of human derived thyroid carcinoma cells proved to be easily established and reproducible; furthermore, the orthotopic models also facilitate the process of metastasis [32,33,34]. While the xenograft models are quite straightforward in that the cells grown and collected are injected into the subcutaneous tissues of immunocompromised mice, the orthotopic model is more complex to establish, since it requires a microsurgical procedure to directly place the cells into one or both the thyroid lobes [32,33,34]. Nonetheless, since the development of such a model [35], orthotopic implantation has been widely adopted to study the genetic and bio-molecular patterns of thyroid carcinogenesis [9,36,37,38,39,40,41,42,43,44,45,46,47,48].

Recently, our group developed a high-frequency ultrasound (HFUS) guided procedure for the orthotopic implantation of FTC cells, demonstrating its feasibility and comparing tumor growth curves with the standard surgical procedure, showing interesting results (see below) [34]. In any case, the main limitations of such in vivo models are the use of mice with various degrees of immunodeficiency, which precludes studying the interactions between the cancer cells and the host immune system [42] and hence the ability to predict the efficacy of clinical therapeutics.

Finally, patient-derived tumor xenograft (PDTX) is a model in which the primary tumor of the patient, obtained surgically is directly transferred into an immunodeficient mouse [49]. Fresh surgical tissue is sectioned into approximately 3 mm^3^ sections, followed by subcutaneous or orthotopic implantation. The PDTX model shows a strong similarity to the parental tumor and allows for the study of the tumor microenvironment and the interaction between tumor cells and their stroma [49].

### 2.3. Metastatic Mouse Model of Thyroid Cancer

Even if the orthotopic model closely resembles the original biological tumor’s behavior in term of local invasion and metastasis [35,36,37,40], particularly when aggressive ATC cells are used, other specific models have been developed to study metastasis [32,45]. Furthermore, in the orthotopic mouse models, the local invasion of neck tissues by the primary tumor can lead to animal suffering, due to the inability to swallow or breath properly; thus, there might not be enough time for the development of metastasis [39,40]. Hence, direct injection into the bloodstream via the tail vein or into the left ventricle allows the dissemination of the selected cell line to all organs, with varying degrees of attachment in the lungs, bones, brain, liver, lymph nodes, etc. [42,45]. Metastasis localization and the tumor growth rate depend on the cell line, on the ability and the determination of investigators to look for them and on the mouse strain, i.e. on the level of immunodepression [32,42].

## 3. In Vivo Imaging for the Molecular Characterization of Thyroid Carcinoma Mouse Models

Almost all preclinical imaging techniques have been used, either alone or in combination to characterize the molecular features of differentiated, poorly differentiated and anaplastic mouse models of thyroid carcinomas. Following is a review of recent papers that have investigated these different modalities and in Table 1 includes a summary of all the studies included.

### 3.1. Nuclear Imaging

Over the past several decades, nuclear medicine has played a central role in both imaging and therapy of human thyroid cancer patients [64,65]. Indeed, the ability to target the sodium/iodide symporter (NIS) with radioiodine has been the basis for nuclear medicine and therapy in human thyroid carcinoma [50].

The NIS is an integral plasma membrane glycoprotein localized in the basolateral membrane that mediates active iodine (I^−^) transport from the extracellular fluid along with two sodium ions into benign or malignant thyroid follicular cells [54,66,67]. Radioisotopes of iodine, i.e., ^124^I and ^131^I, have been used for decades to image thyroid tumors with single photon emission tomography (SPECT) [50]. Nonetheless, thyroid SPECT imaging has some limitations, such as poor resolution and sensitivity in detecting small volume lesions and the long half-lives for radioiodine isotopes leading to superfluously high radiation doses [50]. Hence, over the years, specific positron emission tomography (PET) tracers have been researched, due to the better sensitivity and specificity of this technique compared to SPECT. Tetrafluoroborate (TFB) is a molecule that binds the NIS and is specifically collected into the thyroid, hence the feasibility of radio labelling it with ^18^F. Its ability to trace NIS expression has recently been studied as well. A *TRβ^PV/PV^* transgenic mouse and a control BALB/C wild type mouse have been imaged with a dedicated PET/computed tomography (CT) scanner thirty minutes after the intravenous injection of 5 MBq of [^18^F] TFB in 50 µL. The *TRβ^PV/PV^* mouse had a spontaneous large FTC and the PET scan showed a high specific uptake only in the tumor region [50]. On the other hand, the non-tumor bearing BALB/C mouse showed a high uptake in the thyroid and in the stomach, which is also known to express NIS and a lower uptake in the salivary and mammary glands; the excretion was exclusively renal, as demonstrated by the high signal in the urinary bladder [50]. These in vivo results were confirmed by ex vivo biodistribution studies [50].

The NIS has also been studied as a reporter for gene expression in the human pancreatic adenocarcinoma cell line HPAF, confirming the feasibility of PET imaging with radiolabeled sodium iodide (Na^124^I) [68]. One limitation of the study is that NIS imaging does not allow the researcher to distinguish between benign and malignant lesions. In fact, malignant lesions usually do not show iodide uptake, while most of the “cold” nodules are benign formations.

Another interesting molecule expressed by thyroid carcinomas is galectin-3, which has been demonstrated to be overexpressed in more than 94% of thyroid carcinomas, excluding the MTC, while benign thyroid proliferations, such as nodular hyperplasia and adenoma, do not express this marker [52]. The FRO82-1 (ATC), WRO82-1 (FTC) and BCPAP (PTC) cells, all confirmed to express galectin-3 in vitro, were subcutaneously injected in the right thigh (1.0–1.5 × 10^7^ cells in 100 µL PBS) in female athymic Nude-Foxn1nu/nu mice, 5 weeks old. A 60 min static PET/CT acquisition was performed 48 h after intravenous injection of 1.5 MBq (40 µCi) of ^89^Zr-desferrioxamine-thioureyl-phenyl-isothiocyanate (DFO) rat monoclonal antibody against galectin-3. The results showed a high uptake of the tracer in all tumors and no uptake in the thyroid gland, which was considered as the negative control. Moreover, the specificity of the tracer was confirmed with 100-fold excess of unlabeled monoclonal antibody pre-injection [52].

To enhance early diagnosis of MTC, a radiolabeled single-chain fragment of variable (scFv) antibody anti-MTC has been studied with SPECT/CT [53]. Nude mice bearing TT (MTC) cell line xenografts were intravenously injected with ^131^I-scFv and scanned at 12 h and 1, 2 and 3 days post injection after sealing the normal thyroid with potassium iodide [53]. A high activity was demonstrated in the tumor tissue as soon as 12 h post injection but the background signal remained relatively high until 1 day post injection, therefore delaying the best time for SPECT acquisition to 2 to 3 days post injection.

Nuclear imaging has also been used to evaluate gene expression effects on MTC [54]. Indeed, the *N-myc downstream-regulated gene* (*NDRG*) 2 has been considered a candidate tumor suppressor gene. The TT (MTC) cell line was transfected with a lentivirus to obtain a stably transduced cell overexpressing *NDRG2* [54]. Xenografts were obtained by injecting 5 × 10^6^ cells subcutaneously into *nu*/*nu* mice [54]. When the tumors reached 10 mm in diameter, the mice were intraperitoneally injected with 1 mCi ^99m^TcO_4_^−^ and SPECT images were taken after 10 min [54]. This study demonstrated that TT-NDRG2 cells grew slowly and the mean volume of the tumors was lessened by 60% compared with the controls. This study also showed that MTC cells acquired the ability to accumulate ^99m^TcO_4_^−^ after *NDRG2* gene expression [54].

The PTC and FTC are highly vascularized and have been reported to respond well to anti-angiogenic therapies [51]. Endothelin (ET) is a vasoactive peptide that enhances angiogenesis through vascular-endothelial growth factor (VEGF) stimulation; hence, its receptors (ET_A_R and ET_B_R) can be used as molecular targets for imaging thyroid tumors [51]. Xenografts of the PTC K1 cell line were obtained with the subcutaneous injection of 5 × 10^6^ cells in 200 µL of phosphate-buffered saline (PBS) in 10–12 week old athymic nude *nu*/*nu* mice [51]. Two to 3 weeks after this procedure, 60 min dynamic PET scans were performed using a small-animal PET rodent model scanner after the intravenous injection of 3 to 10 MBq of [^18^F]-glycosilated-PD156707, a non-peptide ET_A_R specific ligand [51]. The in vivo results showed uptake of the radiotracer in the tumor tissue and in healthy myocardium as well as in the bile and intestines, due to its hepatobiliary excretion and rapid fast blood clearance. Nonetheless, due to the tracer hydrophobicity and the low tumor uptake, the clinical translation of this tracer was deemed highly unfavorable [51].

Cerenkov radiation is generated when charged particles travel with a velocity at a speed greater than the phase velocity of light in the given medium; the radioisotopes ^131^I and ^124^I showed sufficient energy to result in Cerenkov radiation that can be eventually visualized with optical imaging equipment [55,56]. BALB/C male mice were treated to induce either hypothyroidism or hyperthyroidism, or took as control and the thyroid function was assessed by measuring the serum total thyroxin. Once the models were established, 18.5 MBq of ^131^I were intraperitoneally injected and each mouse was studied with γ-scintigraphy and Cerenkov luminescence imaging (CLI) 24 h later. The CLI results showed a reduction or increase of luminescence signal in the hypo- and hyper-thyroidal mice, respectively and such result was confirmed by γ-scintigraphy [55]. In a different study, the ability of CLI to detect luminescent signals in mice bearing NIS-expressing cells xenograft injected with ^124^I has been demonstrated as well [56].

### 3.2. Optical Imaging

Optical imaging techniques have been widely used in thyroid carcinoma research. Also with optical imaging, it is still difficult to differentiate between benign and malignant thyroid nodules. Matrix metallo-proteases (MMPs) have been suggested as possible biomarkers to reach this aim [57]. To study the expression levels of MMPs, a MMP activatable photoacoustic probe was designed with preferential cleavage of MMP-2 and MMP-9 and with the use of a fluorescent molecule [57]. Photoacoustic (PA) tomography is an optical imaging method usually combined with ultrasonography, which identifies the acoustic response of laser-excited molecules [69]. In mouse xenografts of FTC-133 (an FTC cell line), different imaging protocols were applied. Fluorescence molecular tomography (FMT) and PA imaging were performed after either intravenous or intratumoral injection of the probe. Next, a continuous PA acquisition of 140 min after intravenous injection of the probe was performed for two wavelengths (680 and 750 nm), since the signal difference between these two wavelengths represents the cleavage of the probe [57]. The results from these experiments confirmed the high expression of MMP levels, particularly of MMP-9 in FTC-133 tumors in vivo with both PA and FMT imaging, due to the specificity of the obtained signals [62]. Since PA offers a greater spatial resolution, signal specificity and depth of penetration, it has been advocated as a future noninvasive method for FTC diagnosis [57,69].

The MMP-9 plays an important role in PTC biology and therefore a fluorescently labeled MMP-9 ratiometric activatable cell-penetrating peptide (RACPP) was developed for the molecular targeting of PTC [58]. A transgenic BRAF V600E murine model for spontaneous PTC was used in this study; 10 nanomoles in 50 µL of RACPP were injected retro-orbitally, followed by a 2 h wash-out period [58]. Then, the mice underwent to surgical excision of the thyroid under white-light illumination and the surgical field was visualized with a customized ratiometric fluorescence imaging-dissecting microscope [58]. This study showed that the fluorescent clean-up phase did not significantly prolong the surgical time and in 5 of the 7 animals studied with this system, suspicious fluorescent areas were detected after cancer removal; 2 of the animals had positive results for PTC, 2 were negative and 1 suspicious area was not large enough for histology. These results support the use of RACPP fluorescence-guided surgery as a potential tool in oncologic surgical procedures involving differentiated thyroid cancers [58].

Fluorescence and bioluminescence imaging techniques have been widely used to follow thyroid carcinoma growth rates [40]. The following thyroid cancer cell lines were all stably transfected with an engineered plasmid to obtain the simultaneous expression of both luciferase and enhanced GFP: 8505C, CAL62, BCPAP, SW1736, C643, HTh7, HTh74, TPC-1, MDA-T41, T238, K1/GLAG-66 and THJ-16T. For each cell line, 5 × 10^5^ cells re-suspended in 5 μL of PBS were orthotopically injected into the right thyroid lobe; after injection of 3 mg of d-luciferine in 200 µL, each mouse was imaged weekly using bioluminescence [42]. Tumor volumes were measured with a caliper. For the metastatic models, 1 × 10^5^ thyroid cancer cells were injected into the left ventricle of athymic nude mice and the same imaging protocol was applied [42]. This experiment allowed the researchers to non-invasively determine the uptake rate, growth curves and metastatic ability of a wide panel of thyroid carcinoma cell lines [42].

In a similar study, the WRO cell line, established from the metastases of a FTC patient, the TT2609-CO2 cell line, established from a primary FTC and the FTC-238 cell line, established from a lung metastasis, were stably transfected with a vector expressing the firefly luciferase gene [45]. 5 × 10^5^ cells of the described transfected cell lines were re-suspended in 10 μL Matrigel/Roswell Park Memorial Institute (RPMI) or Dulbecco’s Modified Eagle’s Medium (DMEM) at 1:1 dilution and were orthotopically injected into the right thyroid gland of non-diabetic obese (NOD) SCID gamma (NSG) mice. The mice were imaged weekly with bioluminescence dedicated hardware after the injection of 150 mg/kg of d-luciferin [45]. The WRO cell line was found to be the more aggressive, with a growth rate 2 to 4 times higher than the other cell lines and the mice did not survive 14 to 18 days after the injection. However, TT2609-CO2 cells showed a delayed onset and mice injected with this cell line had a total survival time of 57 to 70 days [45]. Further evidence of WRO aggressiveness was the development of spontaneous pulmonary metastases after the orthotopic implantation, whereas TT2609-CO2 and FTC-238 were not able to produce metastases, even after intravenous injection [45].

**Figure 1:**
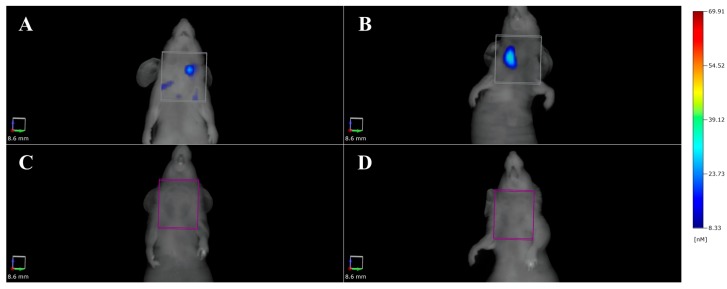
Fluorescent molecular tomography (FMT) of orthotopic ATC bearing mice injected with a 750 nm dye labeled with anti-CD44 antibody (**A**,**B**) or with the unlabeled dye (**C**,**D**). In (**A**): 2 h after injection of the anti-CD44 labeled dye, diffuse signal is present in the neck region, with some uptake in the normal thyroid lobe. In (**B**): after 6 h, the probe is specifically concentrated into the neoplastic lobe. In (**C**) and (**D**): the control mouse injected with the unlabeled probe and 2 and 6 h after the injection, respectively; no signal was identified in the neck region.

Fluorescent molecular tomography (FMT) has also been used by our group to quantify the expression of the CD44 receptor in an orthotopic mouse model of ATC. The model was obtained by the HFUS-guided intra-thyroidal injection of 8505C cells in BALB/C nude mice. A dedicated 750 nm wavelength dye was labeled with anti-CD44 monoclonal antibody and 100 µL of the saline-reconstituted solution containing the probe was injected intravenously. Fluorescence imaging was then performed after 2, 6 and 24 h post injection. As shown in Figure 1 a specific signal was obtained.

### 3.3. High Frequency Ultrasound

**Figure 2:**
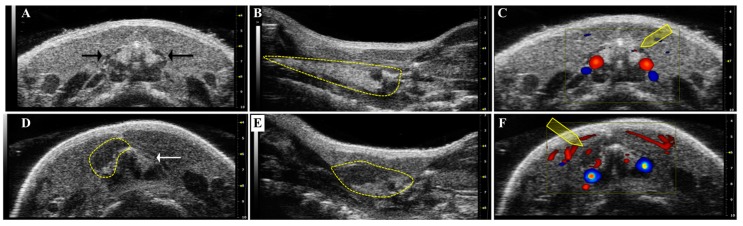
HFUS of the neck region of normal (**A**–**C**) and of orthotopic ATC bearing mouse (**D**–**F**). In (**A**): trans-axial brightness (B-)mode scan of the neck region at the level of the first tracheal rings; black arrows point to the normal thyroid lobes. In (**B**): longitudinal B-mode scan paramedian to the trachea; the yellow dotted line identifies a normal thyroid lobe. In (**C**): trans-axial color-Doppler scan of (**A**); the yellow arrow indicates the low blood flow of the normal thyroid. In red, blood flow towards the ultrasound beam, in blue, blood flow shifting away from it. In (**D**): trans-axial B-mode scan of the neck region at the level of the first tracheal rings; white arrow points to the normal thyroid lobe and the yellow dotted line identifies the orthotopic ATC two weeks after injection. In (**E**): longitudinal B-mode scan paramedian to the trachea; the yellow dotted line identifies the orthotopic ATC two weeks after injection. In (**F**): trans-axial color-Doppler scan of (**D**); the yellow arrow indicates the high blood flow of the orthotopic ATC two weeks after injection. In red, blood flow towards the ultrasound beam, in blue, blood flow shifting away from it.

High frequency ultrasound (HFUS) is the gold standard morphologic technique for a precise thyroid structure evaluation and a tumor volume calculation. Figure 2 includes HFUS images we have obtained in our laboratory of the neck region of a normal mouse and an orthotopic ATC mouse.

Furthermore, HFUS allows functional imaging through the performance of a qualitative characterization of the vascularization and a molecular and functional preclinical imaging due to the use of the contrast agent. The contrast agent could be used both to enhance the vascular signal targeted to specific marker of diseases as well as to perform a quantitative analysis of specific molecules [34,59,60]. Since in humans, ultrasound is the first line diagnostic screening used to detect thyroid nodules, the translatability of the results of this preclinical tool seems straightforward. Indeed, the ability of HFUS to characterize the morphological features of thyroid lesions has been confirmed [59]. In an initial study, The *Tg-TRK-T1* transgenic mouse strain, which develops PTC, was analyzed weekly by HFUS, as well as a model of thyroid hyperplasia developed by administering propylthiouracil to C57BL/6 mice [59]. The HFUS evaluation allowed for the detection of the normal thyroid in the subhyoid region and the diffuse enlargement of the gland in the propylthiouracil-treated mice [59]. The technique allowed for the detection of 19 nodules in the Tg-TRK-T1 mice, of which 11 were deemed to be malignant; the histological analysis could identify 17 of the HFUS-detected 19 nodules and malignancy was confirmed in 10 out of 11 [65]. In summary, the HFUS technique showed a sensitivity of 100% and a specificity of 60% in detecting nodules and a sensitivity of 91% and a specificity of 86% in diagnosing malignancy [59].

Since then, the HFUS has been used for the detection of different transgenic mice harboring mutations linked to the development of thyroid lesions [60]. The *Rb*^+/−^, the *BRAF* and the *TRβ^PV/PV^* mouse strains were examined weekly by HFUS to evaluate if a thyroid enlargement/lesion was detectable [60]. Once a lesion was identified, the mice underwent ex vivo quantitative ultrasound (US), a technique based on US backscatter that is potentially able to differentiate between benign and malignant lesions and the results showed some correlation with the histological analysis [60].

The expression of tumor angiogenesis receptors for the VEGF was also described in a thyroid cancer mouse model with the aid of targeting contrast agents [61]. Contrast agents for HFUS are essentially micro-bubbles (MB) of gas filled microspheres enveloped by a shell of lipid, protein or polymers that may be decorated with a targeting ligand [61,70]. The *Tg-TRK-T1* transgenic mouse strain was analyzed with contrast enhanced HFUS every 6 months; streptavidin-coated MB dissolved in sterile saline were mixed with biotinylated anti-mouse VEGF receptor 2 (VEGFR2) antibodies and 20 µL of the final solution containing 3.8 × 10^7^ MB was injected intravenously [61]. After a 4 min biodistribution time to allow for MB binding and the wash-out of unattached MB, a destruction pulse was applied and pre- and post-contrast images were processed for quantitative analysis of the receptor expression [61]. The relative measures of VEGFR2 over-expression were significantly higher in thyroid tumors compared to benign nodules as well as to normal thyroid and a significant correlation with ex vivo immunohistochemical evaluation of the studied thyroids further confirmed the ability of this technique [61].

Finally, HFUS has been used by our group to develop a new method for orthotopic implantation of thyroid carcinoma cells [34]. The FTC-133 cell line, 2 × 10^6^ cells in 20 µL, were injected into the right thyroid lobe of 6 week old female BALB/C nude mice either with the standard surgical technique or using the HFUS-guided protocol. The latter showed the advantage of being less invasive, since the injecting needle was guided through the skin into the thyroid lobe without the need for an open surgical field. The mice underwent an HFUS analysis weekly for the measurement of tumor volume and for a qualitative vascular characterization using Color-Doppler integrated software. Compared with the surgical technique, the new procedure was found to be more precise for FTC-133 cells implantation in only one thyroid lobe. As a result of this approach, we had a higher rate of pulmonary metastases detected with CT scans and histology and a longer mouse survival time, demonstrating how the technique was less invasive than the surgical method used conventionally to induce thyroid carcinoma [34].

### 3.4. Magnetic Resonance Imaging

In the last decades, magnetic resonance imaging (MRI) has been gaining great importance for its great soft tissue contrast, the absence of ionizing radiation and the ability to acquire several functional information, by using dedicated sequences [71,72]. Hence, MRI is used not only for its ability to define lesions with great spatial resolution and anatomic detail, or to evaluate vascularization with dynamic contrast enhanced (DCE) studies [40] but also to recover quantitative features that might be able to predict the biological behavior of cancer, in term of response to therapy as well as of metastatic evolution. In this perspective, diffusion weighted (DW) MRI has the ability to evaluate the microscopic mobility of water molecules, which is influenced by environment, i.e., in oncologic lesions, by the cellularity, the presence of necrosis, fibrosis, etc. [71]. The degree of mobility is expressed quantitatively with the apparent diffusion coefficient (ADC) that results reduced in malignant lesions [71]. To date, this approach has been used in preclinical research for the study of the normal brain and cerebral tumors, as well as to predict tumor response to chemotherapies in nasopharyngeal carcinoma and osteosarcoma mouse models, since ADC variations (i.e., increases) appear as soon as one to two days post therapy, much earlier than tumor volume reduction [73,74].

In clinical oncology, ADC values from benign nodules have been demonstrated to be significantly higher than malignant ones, in a total of 86 patients compared to 20 healthy control subjects [75]. Furthermore, mean ADC values were significantly different between FTC, PTC and undifferentiated thyroid carcinoma, whereas the ADC maximum values resulted positively correlated with some histopathological indexes, i.e. cell count and total nuclei area, in a clinical series of 20 patients [76]. Nonetheless, the correlation between ADC with Ki-67 index and p53 expression, which are a marker of cell proliferation and a tumor suppressor, respectively, did not reach statistical significance [76]. Hence, in an attempt to improve the efficiency and the reliability of data obtained with diffusion MRI, diffusion kurtosis imaging (DKI) was used to study 58 patients bearing various benign and malignant thyroid lesions [77]. The quantitative parameters obtained, i.e. diffusion coefficient (D) and diffusion kurtosis (K), were significantly different between malignant and benign lesions and D and ADC showed a negative correlation with cell density and D alone with VEGF cells. Moreover, K showed the highest sensitivity and ADC the highest specificity in discriminating benign and malignant thyroid lesions [77]. Anyhow, further investigations involving DW-MRI and DKI parameters in selected histotypes of thyroid carcinoma should be performed in mouse models of thyroid cancer for their future standard clinical application and interpretation.

### 3.5. Multimodal Imaging

Multimodal imaging relies on the ability of combining multiple in vivo molecular imaging technologies to implement the information obtainable with each single technique [78]. This approach has also been applied in the field of thyroid cancer preclinical research. To study the expression of ET_A_R, two fluorescent imaging techniques were applied, namely fluorescent reflectance imaging (FRI) and FMT [41]. The specific non-peptidic ligand PD156707 was labeled with cyanine (Cy) 5.5-*N*-hydroxysuccinimidyl ester for fluorescent imaging; the PTC K1 cell line, 1 × 10^6^ cells in 50 µL, was injected subcutaneously into the right hemithorax or orthotopically into the right thyroid gland of CD-1 nude mice and was allowed to grow [41]. For in vivo imaging, 2.0 nmol of Cy5.5-labeled tracer dissolved in 100 µL of saline was injected intravenously and images were captured up to 48 h post injection [41]. The tumor region showed a high fluorescent signal at FRI as early as 30 min post-injection and an optimal tumor to background contrast was detected at 24 h in the xenograft model. These results were confirmed by FMT, as well; nonetheless, lower signal intensity in the orthotopic model, probably due to the deeper location of the thyroid gland and a more diffuse signal distribution were detected. Of note, in this study, in vivo HFUS analysis was used to follow orthotopic tumor growth prior to in vivo fluorescence studies and ex vivo autoradiographic and real-time polymerase chain reaction analyses [41].

Multimodal imaging was applied to study the effect of a tyrosine kinase inhibitor of the epithelial growth factor receptor (EGFR) and of VEGFR2, namely vandetanib, as anti-thyroid carcinoma therapy [40]. The ATC 8505C and Hth83 cell lines were stably transfected with the luciferase gene (8505C-*lucif* and Hth83-*lucif*) and 5 × 10^5^ or 2.5 × 10^5^ cells, respectively, were orthotopically injected into the right thyroid lobe of athymic nude mice. The mice were then treated with 50 mg/kg vandetanib daily by oral gavage for 4 weeks (8505C-*lucif*) or with 25 mg/kg daily for 3 weeks (Hth83-*lucif*); 5 min before the weekly bioluminescence studies, the mice were injected with 150 mg/kg of d-Luciferine [40]. The bioluminescent signal was used as a surrogate of tumor growth and the results showed a significant reduction of mean tumor volume in both cell lines for vandetanib-treated mice compared to vehicle-treated control mice [40]. A different subset of mice orthotopically injected with 1 × 10^5^ Hth83-*lucif* cells underwent dual-tracer DCE-MRI prior to and after intravenous injection of 0.2 mM (Gd)/kg of PG-Gd-DTPA, a blood-pool contrast agent, after a further 5 min of 0.2 mM/kg of Gd-DTPA [40]. Vascular permeability and vascular volume fractions were calculated from DCE-MRI parametric maps and significant reductions of these parameters were detected compared to the control group, thus explaining the early molecular events through which vandenatib probably exerts its anti-tumor effects [40].

### 3.6. Theranostic

Theranostic is a relatively new and very interesting branch of imaging, which aims to anticipate treatment at the time of the diagnosis, improving the response and minimizing systemic side effects, by combining contrast agents or imaging tracers with therapeutic molecules, or by exploiting tracers’ intrinsic physicochemical characteristics [62,70]. In particular, near-infrared (NIR) fluorescent imaging agents have the ability to generate heat, thus killing cancer cells via hyperthermia when stimulated by light of the appropriate wavelength [62]. 2 × 10^6^ TT (MTC) cells re-suspended in 200 µL of PBS were injected subcutaneously into athymic *nu*/*nu* nude of 4–6 weeks of age. When the tumor mass reached 0.5 cm in diameter, the fluorescent probe, IR820 labeled with amino-glucose (AG) for glucose-transporter 1 targeting, was intravenously injected at 10 mg/kg and images were collected after 2, 4, 6, 12 and 24 h post injection [62]. For hyperthermia induction, the AG-IR820 was intravenously injected at 15 mg/kg, with or without heat sensitizers inhibiting the heat shock protein 70 (Quercetin 0.20 mg in 200 µL per mouse) and the tumor underwent laser exposure at 808 nm and 8 W/cm^2^ for five minutes [62]. Fluorescence imaging confirmed the targeting ability of the AG-IR820, which was further confirmed by the free AG injection prior to the probe administration and the lower relative tumor volume confirmed the antitumor efficacy of the hyperthermia induced by AG-IR820, further enhanced by quercetin administration [62].

Nanoparticles (NPs) represent a promising tool for in vivo multimodality imaging as well as for theranostic applications [43,53,70]. NIR fluorescent polymeric NPs delivering silencing RNA (siRNA) have been studied in a mouse model of ATC; the ATC BRAF V600E-mutated 8505C cell line was used to develop either a subcutaneous xenograft (2 × 10^6^ cells in 200 μL of 50% Matrigel and serum-free culture medium, 1:1 *v*/*v*%) in athymic nude mice or an orthotopic surgical model (5 × 10^5^ cells re-suspended in 10 μL of serum-free RPMI medium) in SCID mice [44]. Mice were treated with 600 µg siRNA loaded NPs per kg every other day for 3 doses (xenograft) or 5 doses (orthotopic) and the mice were imaged 24 h after intravenous injection of a single NP dose [44]. The results showed that the therapy slowed down the tumor growth both in the xenograft and the orthotopic models and in the latter, it reduced the number of pulmonary micro-metastases; fluorescence imaging confirmed the tumor accumulation of NPs and their persistence in the bloodstream up to 24 h after the injection [44].

In the perspective of multimodal imaging mediated by NPs, gold nanoclusters (AuNCs) have been loaded with an NIR probe and iodine (AuNCs@BSA-I) to obtain a fluorescent/CT multimodal platform for a precise ATC diagnosis [63]. Surgically resected human thyroid cancer specimens from a poorly differentiated PTC were cut into 1 mm^3^ pieces within 24 h from resection and implanted into one side of the thyroid of athymic nude mice. In vivo fluorescent imaging showed the ability to distinguish between the normal and the cancerous thyroid lobe based upon the trend of the signal-to-background ratio, since the normal thyroid tissue showed a “fast in, slow out” uptake, whereas the thyroid tumor displayed a “slow in, fast out” pattern [63]. The same pattern was confirmed with CT analysis, thus endorsing the ability of AuNCs@BSA-I to differentiate malignant thyroid carcinoma from normal thyroid based on the biodistribution pattern [63].

The loading capacity of NPs and the possibility to include several molecules in their structure allow potential multi-therapy and multimodal imaging as well. In an orthotopic ATC model obtained with the injection in the right thyroid lobe of athymic nude mice of 5 × 10^5^ Hth83-*lucif* cells and polyethylene glycol (PEG) coated copper sulfide [^64^Cu] CuS, NPs were injected intratumorally and then the mice underwent a PET scan [43]. ^64^Cu is a beta and an Auger emitter, with well described cytotoxic effects in in vivo tumor models in the range of several hundred µm. Therapy was provided either with radiotherapy alone or by phototermal therapy after the intratumoral injection of non-radioactive PEG-CuS NPs and irradiation with a NIR laser, or by the combination of the two, by injecting PEG-[^64^Cu]CuS NPs and NIR laser irradiation [43]. Bioluminescence studies, provided by the transfection of the luciferine gene in the Hth83-*lucif* cell line, provided confirmation of tumor growth in the thyroid, while PET scans allowed for detecting the retention of the NPs within the tumor for up to 48 h; the therapeutic protocol showed all good efficacy compared with the control, with phototermal therapy alone providing reductions in tumor volume of 50%, radiotherapy alone inducing a reduction of 75% and the combination of the two showing a reduction of up to 83% [43]. The main limit of such an interesting theranostic platform would be that, after intravenous administration, only ~6% of the injected dose was concentrated in the tumor at 24 h [43].

## 4. Conclusions

In recent years, significant advances have been made to understand thyroid cancer biology. Due to the development of next generation sequencing techniques, almost all of the genetic alterations responsible for thyroid cancers have been depicted and information is rapidly being translated into the clinical setting.

In this context, preclinical imaging of thyroid cancer mouse models is becoming very important, not only to shed light on the thyroid cancer biology but also for theranostic purposes.

Future research will also be improved with CRISPR/Cas9 genome editing technology. Through this system, it is possible to introduce specific chromosomal rearrangements in vivo by exploiting the DNA damage repair pathway [79]. To date, increasing numbers of mouse models of human cancers have been generated through the use of the CRISPR-Cas9 system to monitor novel treatment strategies in vivo [79,80].

Future research should also focus on finding novel fluorescent and bioluminescent tracers as well as novel integrated imaging modalities with high sensitivity and specificity that can visualize mouse thyroids.

## Figures and Tables

**Table 1:** Preclinical imaging techniques applied in mouse models of thyroid carcinoma.

Imaging	Tracer	Model (Transgene, Cell Line, Xenograft or Orthotopic Implantation)	Histotype	Focus	Reference
PET	[^18^F]-TFB	Transgenic *TRβ^PV/PV^*	FTC	NIS	[50]
PET	[^18^F]glyPD156707	K1—Xenograft	PTC	ET_A_R	[51]
PET	^89^Zr-DFO-mAb	FRO82-1—Xenograft WRO82-1—Xenograft BCPAP—Xenograft	ATC FTC PTC	Galectin-3	[52]
SPECT	^131^I	TT—Xenograft	MTC	Anti-MTC antibody	[53]
SPECT	^99m^TcO_4_^−^	TT—Xenograft	MTC	NDRG2	[54]
CLI	^131^I ^124^I	Hypo-, hyper-thyroidism NIS—Xenograft		NIS	[55] [56]
PA & FMT		FTC133—Xenograft	FTC	MMP	[57]
RFM	RACPP	Transgenic BRAF V600E	PTC	MMP-9	[58]
BLI	GFP	Orthotopic (panel)		Tumor growth and metastatization	[42]
BLI	Luciferase	Orthotopic (panel)		Tumor growth and metastatization	[45]
HFUS		Transgenic *Tg-TRK-T1*	PTC	Tumor growth	[59]
HFUS		Transgenic *Rb*^+/−^ Transgenic *BRAF-TRβ^PV/PV^*	MTC PTC FTC	Tumor growth	[60]
HFUS	antiVEGFR2-MB	Transgenic *Tg-TRK-T1*	PTC	VEGFR2	[61]
HFUS		FTC-133—Orthotopic	FTC	Orthotopic implantation	[34]
FRI & FMT & HFUS	Cy5.5-PD156707	K1—Xenograft	PTC	ET_A_R Tumor growth	[41]
BLI & DCE-MRI	Luciferase PG-Gd-DTPA	8505C—Orthotopic Hth83—Orthotopic	ATC ATC	EGFR VEGFR2	[40]
FMT	AG-IR820	TT—Xenograft	MTC	Glucose-transporter 1	[62]
FMT		8505C-BRAF V600E—Xenograft 8505C-BRAF V600E—Orthotopic	ATC	Therapy effect	[44]
FMT & CT	AuNCs@BSA-I	Human derived poorly differentiate PTC—Xenograft	PTC	Differentiating malignant tissues	[63]
PET & BLI	PEG-[^64^Cu]CuS NPs	Hth83—Orthotopic	ATC	Therapy effect	[43]

The table summarizes the imaging techniques applied to study mouse models of thyroid carcinoma, including the tracer used, which models were applied and for which scope and/or target. Abbreviations: PET, positron emission tomography; TFB, tetrafluoroborate; FTC, follicular thyroid carcinoma; NIS, sodium/iodide symporter; PTC, papillary thyroid carcinoma; ET_A_R, endothelin A receptor; DFO: desferrioxamine-thioureyl-phenyl-isothiocyanate; mAb: monoclonal antibody; ATC, anaplastic thyroid carcinoma; SPECT, single photon emission computed tomography; MTC, medullary thyroid carcinoma; *NDRG2, N-myc downstream-regulated gene 2*; PA, photoacoustic imaging; FMT, fluorescent molecular tomography; MMP, matrix metallo-proteinase; RFM, ratiometric fluorescence microscopy; RACPP, ratiometric activatable cell-penetrating peptide; BLI, bioluminescence imaging; RFP, red fluorescent protein; GFP, green fluorescent protein; HFUS, high frequency ultrasound; VEGFR2, vascular endothelial growth factor receptor 2; MB, micro bubbles; FRI, fluorescent reflectance imaging; DCE, dynamic contrast enhanced; MRI, magnetic resonance imaging; DTPA, diethylenetriaminepentaacetic acid; EGFR, epithelial growth factor receptor; CLI, Cerenkov luminescence imaging; AG, amino-glucose; NCs, nanoclusters; PEG, polyethylene glycol; NPs, nanoparticles.

## Abbreviations

ADC	Apparent Diffusion Coefficient
ATC	Anaplastic thyroid carcinoma
BRAF	V-raf murine sarcoma viral oncogene homolog B
CLI	Cerenkov luminescence imaging
CT	Computed tomography
Cy	Cyanine
DCE	Dynamic contrast enhanced
DFO	Desferrioxamine-thioureyl-phenyl-isothiocyanate
DMEM	Dulbecco’s modified Eagle’s medium
DW	Diffusion weighted
EGFR	Epithelial growth factor receptor
ET	Endothelin
FMT	Fluorescence molecular tomography
FTC	Follicular thyroid carcinoma
GFP	Green fluorescent protein
HFUS	High-frequency ultrasound
MAPK	Mitogen-activated protein kinase
miRNA	MicroRNA
MRI	Magnetic resonance imaging
MTC	Medullary thyroid carcinoma
NDRG2	N-myc downstream-regulated gene 2
NIR	Near-infrared
NIS	Sodium/iodide symporter
NK	Natural killer
NOD	non-diabetic obese
NSG	NOD SCID gamma
NTRK1	Neurotrophic Receptor Tyrosine Kinase 1
PA	Photoacoustic tomography
PBS	Phosphate-buffered saline
PDTX	Patients derived tumor xenografts
PET	Positron emission tomography
PTC	Papillary thyroid carcinoma
PTEN	Phosphatase and tensin homolog
RACPP	Ratiometric activatable cell-penetrating peptide
RAS	Rat sarcoma
RET	Rearranged During Transfection
RPMI	Roswell Park Memorial Institute
SCID	Severely combined immune-deficient
SPECT	Single photon emission tomography
STR	Short tandem repeat
TC	Thyroid cancer
TCGA	Cancer Genome Atlas
Tet/O	Tetracycline-inducible mouse model
TFB	Tetrafluoroborate
Tg	Thyroglobulin
THRB	Thyroid hormone receptor-β
TPO-Cre	Thyroid peroxidase-driven cre recombinase
VEGF	Vascular-endothelial growth factor

## References

[1] Siegel R.L., Miller K.D., Jemal A. (2017). Cancer Statistics, 2017. CA Cancer J. Clin..

[2] Kitahara C.M., Sosa J.A. (2016). The changing incidence of thyroid cancer. Nat. Rev. Endocrinol..

[3] Fagin J.A., Wells S.A. (2016). Biologic and Clinical Perspectives on Thyroid Cancer. N. Engl. J. Med..

[4] Dralle H., Machens A., Basa J., Fatourechi V., Franceschi S., Hay I.D., Nikiforov Y.E., Pacini F., Pasieka J.L., Sherman S.I. (2015). Follicular cell-derived thyroid cancer. Nat. Rev. Dis. Primers.

[5] Nikiforov Y.E., Nikiforova M.N. (2011). Molecular genetics and diagnosis of thyroid cancer. Nat. Rev. Endocrinol..

[6] Molinaro E., Romei C., Biagini A., Sabini E., Agate L., Mazzeo S., Materazzi G., Sellari-Franceschini S., Ribechini A., Torregrossa L. (2017). Anaplastic thyroid carcinoma: From clinicopathology to genetics and advanced therapies. Nat. Rev. Endocrinol..

[7] Xu B., Ghossein R. (2016). Genomic Landscape of poorly Differentiated and Anaplastic Thyroid Carcinoma. Endocr. Pathol..

[8] Maia A.L., Wajner S.M., Vargas C.V. (2017). Advances and controversies in the management of medullary thyroid carcinoma. Curr. Opin. Oncol..

[9] Agrawal N., Akbani R., Aksoy B.A., Ally A., Arachchi H., Asa S.L., Auman J.T., Balasundaram M., Balu S., Baylin S.B. (2014). Integrated genomic characterization of papillary thyroid carcinoma. Cell.

[10] Howell G.M., Hodak S.P., Yip L. (2013). RAS mutations in thyroid cancer. Oncologist.

[11] Raman P., Koenig R.J. (2014). Pax-8-PPAR-gamma fusion protein in thyroid carcinoma. Nat. Rev. Endocrinol..

[12] Asa S.L., Ezzat S. (2017). The epigenetic landscape of differentiated thyroid cancer. Mol. Cell. Endocrinol..

[13] Accardo G., Conzo G., Esposito D., Gambardella C., Mazzella M., Castaldo F., Di Donna C., Polistena A., Avenia N., Colantuoni V. (2017). Genetics of medullary thyroid cancer: An overview. Int. J. Surg..

[14] Walrath J.C., Hawes J.J., Van Dyke T., Reilly K.M. (2010). Genetically engineered mouse models in cancer research. Adv. Cancer Res..

[15] Doyle A., McGarry M.P., Lee N.A., Lee J.J. (2012). The construction of transgenic and gene knockout/knockin mouse models of human disease. Transgenic Res..

[16] Rusinek D., Krajewska J., Jarzab M. (2016). Mouse models of papillary thyroid carcinoma—Short review. Endokrynol. Pol..

[17] Kirschner L.S., Qamri Z., Kari S., Ashtekar A. (2016). Mouse models of thyroid cancer: A 2015 update. Mol. Cell. Endocrinol..

[18] Vitale G., Gaudenzi G., Circelli L., Manzoni M.F., Bassi A., Fioritti N., Faggiano A., Colao A. (2017). Animal models of medullary thyroid cancer: State of the art and view to the future. Endocr. Relat. Cancer.

[19] Li D.D., Zhang Y.F., Xu H.X., Zhang X.P. (2015). The role of BRAF in the pathogenesis of thyroid carcinoma. Front. Biosci..

[20] Knauf J.A., Ma X., Smith E.P., Zhang L., Mitsutake N., Liao X.H., Refetoff S., Nikiforov Y.E., Fagin J.A. (2005). Targeted expression of BRAFV600E in thyroid cells of transgenic mice results in papillary thyroid cancers that undergo dedifferentiation. Cancer Res..

[21] Chakravarty D., Santos E., Ryder M., Knauf J.A., Liao X.H., West B.L., Bollag G., Kolesnick R., Thin T.H., Rosen N. (2011). Small-molecule MAPK inhibitors restore radioiodine incorporation in mouse thyroid cancers with conditional BRAF activation. J. Clin. Investig..

[22] Russell J.P., Powell D.J., Cunnane M., Greco A., Portella G., Santoro M., Fusco A., Rothstein J.L. (2000). The TRK-T1 fusion protein induces neoplastic transformation of thyroid epithelium. Oncogene.

[23] Cunha L.L., Marcello M.A., Ward L.S. (2014). The role of the inflammatory microenvironment in thyroid carcinogenesis. Endocr. Relat. Cancer.

[24] Taylor E., Heyland A. (2017). Evolution of thyroid hormone signaling in animals: Non-genomic and genomic modes of action. Mol. Cell. Endocrinol..

[25] Suzuki H., Willingham M.C., Cheng S.Y. (2002). Mice with a mutation in the thyroid hormone receptor beta gene spontaneously develop thyroid carcinoma: A mouse model of thyroid carcinogenesis. Thyroid.

[26] Kato Y., Ying H., Willingham M.C., Cheng S.Y. (2004). A tumor suppressor role for thyroid hormone beta receptor in a mouse model of thyroid carcinogenesis. Endocrinology.

[27] Kaneshige M., Kaneshige K., Zhu X., Dace A., Garrett L., Carter T.A., Kazlauskaite R., Pankratz D.G., Wynshaw-Boris A., Refetoff S. (2000). Mice with a targeted mutation in the thyroid hormone beta receptor gene exhibit impaired growth and resistance to thyroid hormone. Proc. Natl. Acad. Sci. USA.

[28] Velez-Cruz R., Johnson D.G. (2017). The Retinoblastoma (RB) Tumor Suppressor: Pushing Back against Genome Instability on Multiple Fronts. Int. J. Mol. Sci..

[29] Williams B.O., Remington L., Albert D.M., Mukai S., Bronson R.T., Jacks T. (1994). Cooperative tumorigenic effects of germline mutations in Rb and p53. Nat. Genet..

[30] Akeno N., Miller A.L., Ma X., Wikenheiser-Brokamp K.A. (2015). p53 suppresses carcinoma progression by inhibiting mTOR pathway activation. Oncogene.

[31] Harvey M., Vogel H., Lee E.Y., Bradley A., Donehower L.A. (1995). Mice deficient in both p53 and Rb develop tumors primarily of endocrine origin. Cancer Res..

[32] Kim C.S., Zhu X. (2009). Lessons from mouse models of thyroid cancer. Thyroid.

[33] Antonello Z.A., Nucera C. (2014). Orthotopic mouse models for the preclinical and translational study of targeted therapies against metastatic human thyroid carcinoma with BRAF(V600E) or wild-type BRAF. Oncogene.

[34] Greco A., Albanese S., Auletta L., Mirabelli P., Zannetti A., D’Alterio C., Di Maro G., Orlandella F.M., Salvatore G., Soricelli A. (2016). High-Frequency Ultrasound-Guided Injection for the Generation of a Novel Orthotopic Mouse Model of Human Thyroid Carcinoma. Thyroid.

[35] Kim S., Park Y.W., Schiff B.A., Doan D.D., Yazici Y., Jasser S.A., Younes M., Mandal M., Bekele B.N., Myers J.N. (2005). An orthotopic model of anaplastic thyroid carcinoma in athymic nude mice. Clin. Cancer Res..

[36] Ahn S.H., Henderson Y., Kang Y., Chattopadhyay C., Holton P., Wang M., Briggs K., Clayman G.L. (2008). An orthotopic model of papillary thyroid carcinoma in athymic nude mice. Arch. Otolaryngol. Head Neck Surg..

[37] Nucera C., Nehs M.A., Mekel M., Zhang X., Hodin R., Lawler J., Nose V., Parangi S. (2009). A novel orthotopic mouse model of human anaplastic thyroid carcinoma. Thyroid.

[38] Todaro M., Iovino F., Eterno V., Cammareri P., Gambara G., Espina V., Gulotta G., Dieli F., Giordano S., De Maria R. (2010). Tumorigenic and metastatic activity of human thyroid cancer stem cells. Cancer Res..

[39] Tran Cao H.S., Kaushal S., Snyder C.S., Ongkeko W.M., Hoffman R.M., Bouvet M. (2010). Real-time imaging of tumor progression in a fluorescent orthotopic mouse model of thyroid cancer. Anticancer Res..

[40] Gule M.K., Chen Y., Sano D., Frederick M.J., Zhou G., Zhao M., Milas Z.L., Galer C.E., Henderson Y.C., Jasser S.A. (2011). Targeted therapy of VEGFR2 and EGFR significantly inhibits growth of anaplastic thyroid cancer in an orthotopic murine model. Clin. Cancer Res..

[41] Buther K., Compeer M.G., De Mey J.G., Schober O., Schafers M., Bremer C., Riemann B., Holtke C. (2012). Assessment of endothelin-A receptor expression in subcutaneous and orthotopic thyroid carcinoma xenografts in vivo employing optical imaging methods. Endocrinology.

[42] Morrison J.A., Pike L.A., Lund G., Zhou Q., Kessler B.E., Bauerle K.T., Sams S.B., Haugen B.R., Schweppe R.E. (2015). Characterization of thyroid cancer cell lines in murine orthotopic and intracardiac metastasis models. Horm. Cancer.

[43] Zhou M., Chen Y., Adachi M., Wen X., Erwin B., Mawlawi O., Lai S.Y., Li C. (2015). Single agent nanoparticle for radiotherapy and radio-photothermal therapy in anaplastic thyroid cancer. Biomaterials.

[44] Liu Y., Gunda V., Zhu X., Xu X., Wu J., Askhatova D., Farokhzad O.C., Parangi S., Shi J. (2016). Theranostic near-infrared fluorescent nanoplatform for imaging and systemic siRNA delivery to metastatic anaplastic thyroid cancer. Proc. Natl. Acad. Sci. USA.

[45] Reeb A.N., Ziegler A., Lin R.Y. (2016). Characterization of human follicular thyroid cancer cell lines in preclinical mouse models. Endocr. Connect..

[46] Mo J.H., Choi I.J., Jeong W.J., Jeon E.H., Ahn S.H. (2012). HIF-1alpha and HSP90: Target molecules selected from a tumorigenic papillary thyroid carcinoma cell line. Cancer Sci..

[47] Nehs M.A., Nucera C., Nagarkatti S.S., Sadow P.M., Morales-Garcia D., Hodin R.A., Parangi S. (2012). Late intervention with anti-BRAF(V600E) therapy induces tumor regression in an orthotopic mouse model of human anaplastic thyroid cancer. Endocrinology.

[48] Yang Y.J., Na H.J., Suh M.J., Ban M.J., Byeon H.K., Kim W.S., Kim J.W., Choi E.C., Kwon H.J., Chang J.W. (2015). Hypoxia Induces Epithelial-Mesenchymal Transition in Follicular Thyroid Cancer: Involvement of Regulation of Twist by Hypoxia Inducible Factor-1alpha. Yonsei Med. J..

[49] Tentler J.J., Tan A.C., Weekes C.D., Jimeno A., Leong S., Pitts T.M., Arcaroli J.J., Messersmith W.A., Eckhardt S.G. (2012). Patient-derived tumour xenografts as models for oncology drug development. Nat. Rev. Clin. Oncol..

[50] Jauregui-Osoro M., Sunassee K., Weeks A.J., Berry D.J., Paul R.L., Cleij M., Banga J.P., O’Doherty M.J., Marsden P.K., Clarke S.E. (2010). Synthesis and biological evaluation of [^18^F]tetrafluoroborate: A PET imaging agent for thyroid disease and reporter gene imaging of the sodium/iodide symporter. Eur. J. Nucl. Med. Mol. Imaging.

[51] Maschauer S., Michel K., Tripal P., Buther K., Kuwert T., Schober O., Kopka K., Riemann B., Prante O. (2013). Synthesis and in vivo evaluation of an ^18^F-labeled glycoconjugate of PD156707 for imaging ETA receptor expression in thyroid carcinoma by positron emission tomography. Am. J. Nucl. Med. Mol. Imaging.

[52] D’Alessandria C., Braesch-Andersen S., Bejo K., Reder S., Blechert B., Schwaiger M., Bartolazzi A. (2016). Noninvasive In Vivo Imaging and Biologic Characterization of Thyroid Tumors by ImmunoPET Targeting of Galectin-3. Cancer Res..

[53] Liu Q., Pang H., Hu X., Li W., Xi J., Xu L., Zhou J. (2016). Construction of human single-chain variable fragment antibodies of medullary thyroid carcinoma and single photon emission computed tomography/computed tomography imaging in tumor-bearing nude mice. Oncol. Rep..

[54] Yin A., Wang C., Sun J., Gao J., Tao L., Du X., Zhao H., Yang J., Li Y. (2016). Overexpression of NDRG2 Increases Iodine Uptake and Inhibits Thyroid Carcinoma Cell Growth In Situ and In Vivo. Oncol. Res..

[55] Ke C.-C., He Z.-M., Hsieh Y.-J., Huang C.-W., Li J.-J., Hwu L., Chen Y.-A., Yang B.-H., Chang C.-W., Huang W.-S. (2017). Quantitative Measurement of the Thyroid Uptake Function of Mouse by Cerenkov Luminescence Imaging. Sci. Rep..

[56] Jeong S.Y., Hwang M.H., Kim J.E., Kang S., Park J.C., Yoo J., Ha J.H., Lee S.W., Ahn B.C., Lee J. (2011). Combined Cerenkov luminescence and nuclear imaging of radioiodine in the thyroid gland and thyroid cancer cells expressing sodium iodide symporter: Initial feasibility study. Endocr. J..

[57] Levi J., Kothapalli S.R., Bohndiek S., Yoon J.K., Dragulescu-Andrasi A., Nielsen C., Tisma A., Bodapati S., Gowrishankar G., Yan X. (2013). Molecular photoacoustic imaging of follicular thyroid carcinoma. Clin. Cancer Res..

[58] Orosco R.K., Savariar E.N., Weissbrod P.A., Diaz-Perez J.A., Bouvet M., Tsien R.Y., Nguyen Q.T. (2016). Molecular targeting of papillary thyroid carcinoma with fluorescently labeled ratiometric activatable cell penetrating peptides in a transgenic murine model. J. Surg. Oncol..

[59] Mancini M., Vergara E., Salvatore G., Greco A., Troncone G., Affuso A., Liuzzi R., Salerno P., Scotto di Santolo M., Santoro M. (2009). Morphological ultrasound microimaging of thyroid in living mice. Endocrinology.

[60] Lavarello R.J., Ridgway W.R., Sarwate S.S., Oelze M.L. (2013). Characterization of thyroid cancer in mouse models using high-frequency quantitative ultrasound techniques. Ultrasound Med. Biol..

[61] Mancini M., Greco A., Salvatore G., Liuzzi R., Di Maro G., Vergara E., Chiappetta G., Pasquinelli R., Brunetti A., Salvatore M. (2013). Imaging of thyroid tumor angiogenesis with microbubbles targeted to vascular endothelial growth factor receptor type 2 in mice. BMC Med. Imaging.

[62] Zhou L., Zhang M., Fu Q., Li J., Sun H. (2016). Targeted near infrared hyperthermia combined with immune stimulation for optimized therapeutic efficacy in thyroid cancer treatment. Oncotarget.

[63] Chen X., Zhu H., Huang X., Wang P., Zhang F., Li W., Chen G., Chen B. (2017). Novel iodinated gold nanoclusters for precise diagnosis of thyroid cancer. Nanoscale.

[64] Buscombe J.R. (2007). Radionuclides in the management of thyroid cancer. Cancer Imaging.

[65] Buscombe J., Hirji H., Witney-Smith C. (2008). Nuclear medicine in the management of thyroid disease. Expert Rev. Anticancer Ther..

[66] Perron B., Rodriguez A.M., Leblanc G., Pourcher T. (2001). Cloning of the mouse sodium iodide symporter and its expression in the mammary gland and other tissues. J. Endocrinol..

[67] Ahn B.C. (2016). Personalized Medicine Based on Theranostic Radioiodine Molecular Imaging for Differentiated Thyroid Cancer. BioMed Res. Int..

[68] Groot-Wassink T., Aboagye E.O., Glaser M., Lemoine N.R., Vassaux G. (2002). Adenovirus biodistribution and noninvasive imaging of gene expression in vivo by positron emission tomography using human sodium/iodide symporter as reporter gene. Hum. Gene Ther..

[69] Rao B., Zhang R., Li L., Shao J.Y., Wang L.V. (2017). Photoacoustic imaging of voltage responses beyond the optical diffusion limit. Sci. Rep..

[70] Greco A., Albanese S., Auletta L., De Carlo F., Salvatore M., Howard C.M., Claudio P.P. (2017). Advances in molecular preclinical therapy mediated by imaging. Q. J. Nucl. Med. Mol. Imaging.

[71] Padhani A.R., Liu G., Mu-Koh D., Chenevert T.L., Thoeny H.C., Takahara T., Dzik-Jurasz A., Ross B.D., Van Cauteren M., Collins D. (2009). Diffusion-Weighted Magnetic Resonance Imaging as a Cancer Biomarker: Consensus and Recommendations. Neoplasia.

[72] Chen Z.-Y., Wang Y.-X., Lin Y., Zhang J.-S., Yang F., Zhou Q.-L., Liao Y.-Y. (2014). Advance of Molecular Imaging Technology and Targeted Imaging Agent in Imaging and Therapy. BioMed Res. Int..

[73] Cui Y., Zhang C., Li X., Liu H., Yin B., Xu T., Zhang Y., Wang D. (2015). Intravoxel Incoherent Motion Di usion-weighted Magnetic Resonance Imaging for Monitoring the Early Response to ZD6474 from Nasopharyngeal Carcinoma in Nude Mouse. Sci. Rep..

[74] Foroutan P., Kreahling J.M., Morse D.L., Grove O., Lloyd M.C., Reed D., Raghavan M., Altiok S., Martinez G.V., Gillies R.J. (2013). Diffusion MRI and Novel Texture Analysis in Osteosarcoma Xenotransplants Predicts Response to Anti-Checkpoint Therapy. PLoS ONE.

[75] Bozgeyik Z., Coskun S., Dagli A.F., Ozkan Y., Sahpaz F., Ogur E. (2009). Diffusion-weighted MR imaging of thyroid nodules. Neuroradiology.

[76] Schob S., Voigt P., Bure L., Meyer H.-J., Wickenhauser C., Behrmann C., Höhn A., Kachel P., Dralle H., Hoffmann K.-T. (2016). Diffusion-Weighted Imaging Using a Readout-Segmented, Multishot EPI Sequence at 3 T Distinguishes between Morphologically Differentiated and Undifferentiated Subtypes of Thyroid Carcinoma—A Preliminary Study. Transl. Oncol..

[77] Shi R.-Y., Yao Q.-Y., Zhou Q.-Y., Lu Q., Suo S.-T., Chen J., Zheng W.-J., Dai Y.-M., Wu L.-M., Xu J.-R. (2017). Preliminary study of diffusion kurtosis imaging in thyroid nodules and its histopathologic correlation. Eur. Radiol..

[78] Auletta L., Gramanzini M., Gargiulo S., Albanese S., Salvatore M., Greco A. (2017). Advances in multimodal molecular imaging. Q. J. Nucl. Med. Mol. Imaging.

[79] Guernet A., Grumolato L. (2017). CRISPR/Cas9 editing of the genome for cancer modeling. Methods.

[80] Platt R.J., Chen S., Zhou Y., Yim M.J., Swiech L., Kempton H.R., Dahlman J.E., Parnas O., Eisenhaure T.M., Jovanovic M. (2014). CRISPR-Cas9 knockin mice for genome editing and cancer modeling. Cell.

